# *Aureliella helgolandensis* gen. nov., sp. nov., a novel Planctomycete isolated from a jellyfish at the shore of the island Helgoland

**DOI:** 10.1007/s10482-020-01403-8

**Published:** 2020-03-27

**Authors:** Nicolai Kallscheuer, Sandra Wiegand, Christian Boedeker, Stijn H. Peeters, Mareike Jogler, Patrick Rast, Anja Heuer, Mike S. M. Jetten, Manfred Rohde, Christian Jogler

**Affiliations:** 1 Department of Microbiology, Radboud University, Nijmegen, The Netherlands; 2 Institute for Biological Interfaces 5, Karlsruhe Institute of Technology, Eggenstein-Leopoldshafen, Germany; 3 Leibniz Institute DSMZ, Brunswick, Germany; 4 Central Facility for Microscopy, Helmholtz Centre for Infection Research, Brunswick, Germany; 5 Department of Microbial Interactions, Institute of Microbiology, Friedrich Schiller University, Jena, Germany

**Keywords:** Marine bacteria, North Sea, Biotic surfaces, *Pirellulaceae*

## Abstract

A novel planctomycetal strain, designated Q31a^T^, was isolated from a jellyfish at the shore of the island Helgoland in the North Sea. The strain forms lucid white colonies on solid medium and displays typical characteristics of planctomycetal strains, such as division by budding, formation of rosettes, presence of crateriform structures, extracellular matrix or fibre and a holdfast structure. Q31a^T^ is mesophilic (temperature optimum 27 °C), neutrophilic (pH optimum 7.5), aerobic and heterotrophic. A maximal growth rate of 0.017 h^− 1^ (generation time of 41 h) was observed. Q31a^T^ has a genome size of 8.44 Mb and a G + C content of 55.3%. Phylogenetically, the strain represents a novel genus and species in the recently introduced family *Pirellulaceae*, order *Pirellulales*, class *Planctomycetia*. We propose the name *Aureliella helgolandensis* gen. nov., sp. nov. for the novel species, represented by Q31a^T^ (= DSM 103537^T^ = LMG 29700^T^) as the type strain.

## Introduction

*Planctomycetes* is a bacterial phylum displaying exceptional physiological and morphological features (Fuerst 1995; Lage et al. 2019; Staley et al. 1992; Ward 2010; Wiegand et al. 2020). Members of this phylum can be found in a variety of different habitats on earth, while the majority of species characterised so far have been isolated from aquatic environments (Wiegand et al. 2018). Phylogenetically, the phylum *Planctomycetes*, along with *Chlamydiae*, *Verrucomicrobia* and others, forms the PVC superphylum, which is of medical and biotechnological relevance (Calisto et al. 2019; Wagner and Horn 2006). Planctomycetes have large genomes of up to 12.4 Mb and usually 40–50% of the annotated genes are of unknown function (Jogler et al. 2012; Ravin et al. 2018; Wiegand et al. 2020). The taxonomy of the phylum *Planctomycetes* was recently revised (Dedysh et al. 2019). No changes were made to the original division of the phylum into the classes *Candidatus* Brocadiae, *Phycisphaerae* and *Planctomycetia*, while the latter is now further subdivided into the orders *Gemmatales*, *Isosphaerales*, *Pirellulales* and *Planctomycetales*.

Species belonging to the class *Planctomycetia* have been isolated from various marine biotic and abiotic surfaces (Boersma et al. 2019; Bondoso et al. 2014, 2017; Kallscheuer et al. 2020; Peeters et al. 2020; Vollmers et al. 2017), on which they can be highly abundant (Bengtsson and Øvreås 2010). Due to the oligotrophic nature of marine environments, such species are suggested to digest complex carbon substrates, e.g. from biotic surfaces to which they frequently attach (Jeske et al. 2013; Lachnit et al. 2013). The observed dominance of planctomycetal species e.g. on algal surfaces is astonishing given their slow growth compared to other natural competitors in this ecological niche, e.g. members of the ‘*Roseobacter* group’ (Frank et al. 2014; Wiegand et al. 2018). The underlying mechanisms allowing species of the class *Planctomycetia* to compensate for slower growth are not understood, but might include the capability to produce bioactive small molecules (Graca et al. 2016; Jeske et al. 2016; Kallscheuer et al. 2019c), their resistance against several antibiotics (Cayrou et al. 2010; Godinho et al. 2019) and a specialised machinery for the uptake and intracellular digestion of complex polysaccharides. The latter is suspected to be facilitated by unique pili-forming crateriform structures and an extremely enlarged periplasmic space (Boedeker et al. 2017).

In the last decade, novel microscopic techniques and genetic tools for Planctomycetes (Jogler et al. 2011; Jogler and Jogler 2013; Rivas-Marin et al. 2016) allowed for a more detailed analysis of the cell envelope architecture of these bacteria. Planctomycetes were shown to possess peptidoglycan (Jeske et al. 2015; van Teeseling et al. 2015), supporting the assumption that all free-living bacteria have a peptidoglycan cell wall. The cell envelope architecture of Planctomycetes is therefore similar to that of Gram-negative bacteria (Boedeker et al. 2017; Devos 2014). However, the phylum *Planctomycetes* is still exceptional. Characterised members were found to lack canonical divisome proteins including the otherwise universal FtsZ (Jogler et al. 2012; Pilhofer et al. 2008). Members of the class *Phycisphaera*e divide by binary fission, while budding is performed by species in the class *Planctomycetia* (Wiegand et al. 2020).

To extend the current collection of axenic cultures and as a basis to further study the interesting cell biology and metabolism of Planctomycetes here we describe strain Q31a^T^ isolated from a jellyfish close to the island Helgoland in the North Sea. According to the results of our analyses, the strain represents a novel species and genus in the recently proposed family *Pirellulaceae*, order *Pirellulales* in the class *Planctomycetia* (Dedysh et al. 2019).

## Materials and methods

### Isolation of the novel strain and cultivation

For the isolation and cultivation of strain Q31a^T^, M1H NAG ASW medium was used (Kallscheuer et al. 2019a). Strain Q31a^T^ was isolated from a dead common jellyfish (*Aurelia aurita*) found at the shore of Helgoland Island (exact location 54.188 N 7.875 E) on the 5th of June 2013. A piece of the tentacles was cut off and then swabbed over a M1H NAG ASW plate containing 8 g/L gellan gum, 1000 mg/L streptomycin, 200 mg/L ampicillin and 20 mg/L cycloheximide, which was subsequently incubated at 20 °C for three weeks. The 16S rRNA gene of obtained colonies was amplified by PCR and sequenced following an established protocol (Rast et al. 2017). This step was performed in order to check whether isolated strains represent members of the phylum *Planctomycetes*. DNA extraction and genome sequencing are described in a previously published study (Wiegand et al. 2020).

### Determination of pH and temperature optimum

Cultivation for determination of the pH optimum was performed in M1H NAG ASW medium and for ensuring a stable pH 100 mM HEPES was used for cultivations at pH 7, 7.5 and 8. For cultivation at pH 5 and 6 HEPES was replaced by 100 mM 2-(*N*-morpholino)ethanesulfonic acid (MES), whereas 100 mM *N*-cyclohexyl-2-aminoethanesulfonic acid (CHES) served as a buffering agent at pH 9 and 10. Cultivations for determination of the pH optimum were performed at 28 °C. Cultivations for determination of the temperature optimum were performed in standard M1H NAG ASW medium at pH 7.5.

### Microscopy protocols

Phase contrast and field emission scanning electron microscopy were performed as previously described (Boersma et al. 2019).

### Genome information

Genome and 16S rRNA gene sequence of strain Q31a^T^ are available from GenBank under accession numbers CP036298 and MK559992, respectively. Numbers of carbohydrate-active enzymes were obtained from the CAZY database (Lombard et al. 2014). Gene clusters potentially involved in the production of secondary metabolites were determined using antiSMASH 4.0 (Blin et al. 2017).

### Phylogenetic analysis

16S rRNA gene-based phylogeny was computed for Q31a^T^, the type strains of all described planctomycetal species (assessed in January 2020) and all isolates recently described (Boersma et al. 2019; Kallscheuer et al. 2019a, b, d, 2020; Kohn et al. 2019; Peeters et al. 2020; Rensink et al. 2020). The 16S rRNA gene sequences were aligned with SINA (Pruesse et al. 2012) and the phylogenetic inference was calculated with RAxML (Stamatakis 2014) with a maximum likelihood approach with 1000 bootstraps, nucleotide substitution model GTR, gamma distributed rate variation and estimation of proportion of invariable sites (GTRGAMMAI option). Three 16S rRNA genes of bacterial strains from the PVC superphylum but outside of the phylum *Planctomycetes* (GenBank accession numbers AJ229235, KC665948 and NR_027571) were used as outgroup. For the multi-locus sequence analysis (MLSA) the unique single-copy core genome of the analysed genomes (GenBank acc. no. CP036298) was determined with proteinortho5 (Lechner et al. 2011) with the ‘selfblast’ option enabled. The protein sequences of the resulting orthologous groups were aligned using MUSCLE v.3.8.31 (Edgar 2004). After clipping, partially aligned *C*- and *N*-terminal regions and poorly aligned internal regions were filtered using Gblocks (Castresana 2000). The final alignment was concatenated and clustered using the maximum likelihood method implemented by RaxML (Stamatakis 2014) with the ‘rapid bootstrap’ method and 500 bootstrap replicates. Four planctomycetal genomes from different families were used as outgroup. The average nucleotide identity (ANI) was calculated using OrthoANI (Lee et al. 2016). The average amino acid identity (AAI) was calculated using the aai.rb script of the enveomics collection (Rodriguez-R and Konstantinidis 2016) and the percentage of conserved proteins (POCP) was calculated as described (Qin et al. 2014). The *rpoB* nucleotide sequences were taken from publicly available planctomycetal genome annotations and the sequence identities were determined as described (Bondoso et al. 2013). Upon extracting only those parts of the sequence that would have been sequenced with the described primer set, the alignment and matrix calculation was performed with Clustal Omega (Sievers et al. 2011).

## Results and discussion

### Phylogenetic analysis

**Fig. 1:**
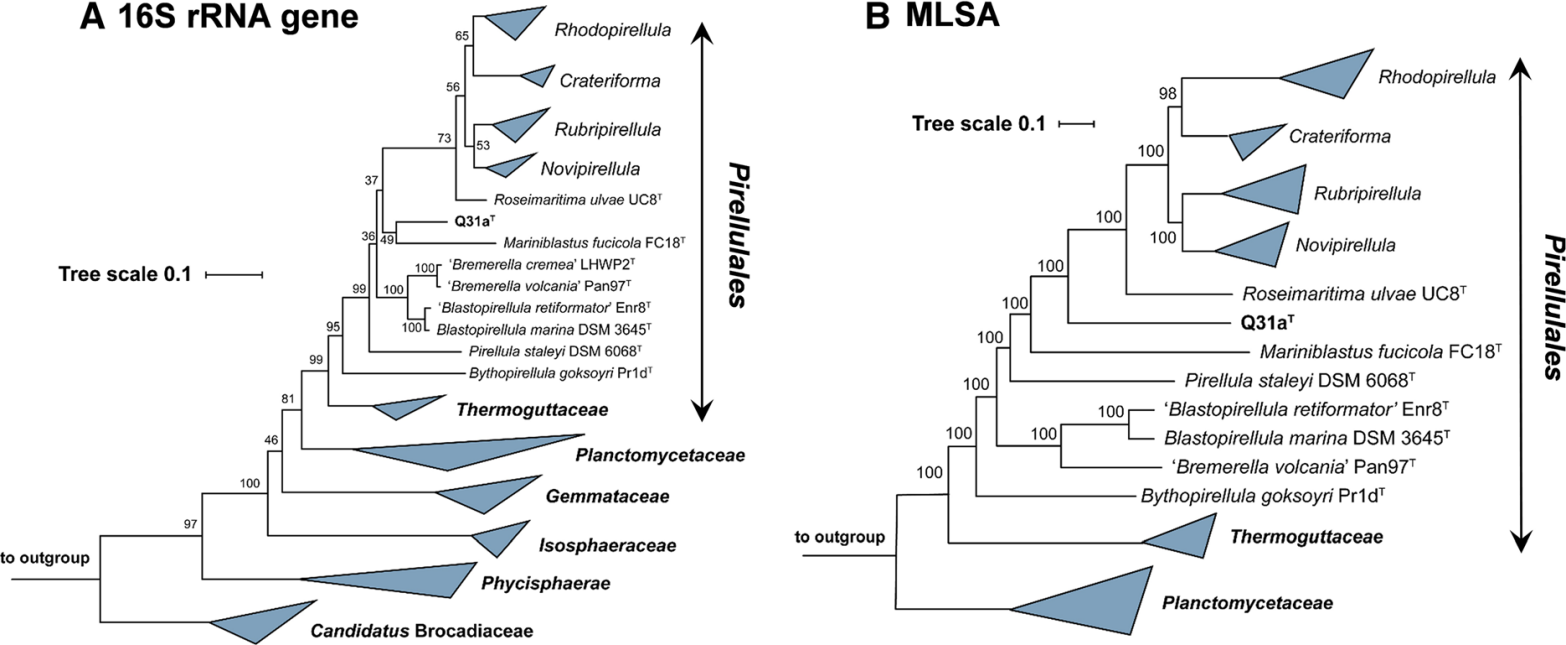
Maximum likelihood phylogenetic analysis. Phylogenetic trees showing the position of strain Q31a^T^. 16S rRNA gene (**a**) and MLSA-based phylogeny (**b**) was computed as described in the "Material and methods" section. Bootstrap values after 1000 re-samplings (16S rRNA gene) or 500 re-samplings (MLSA) are given at the nodes

**Fig. 2:**
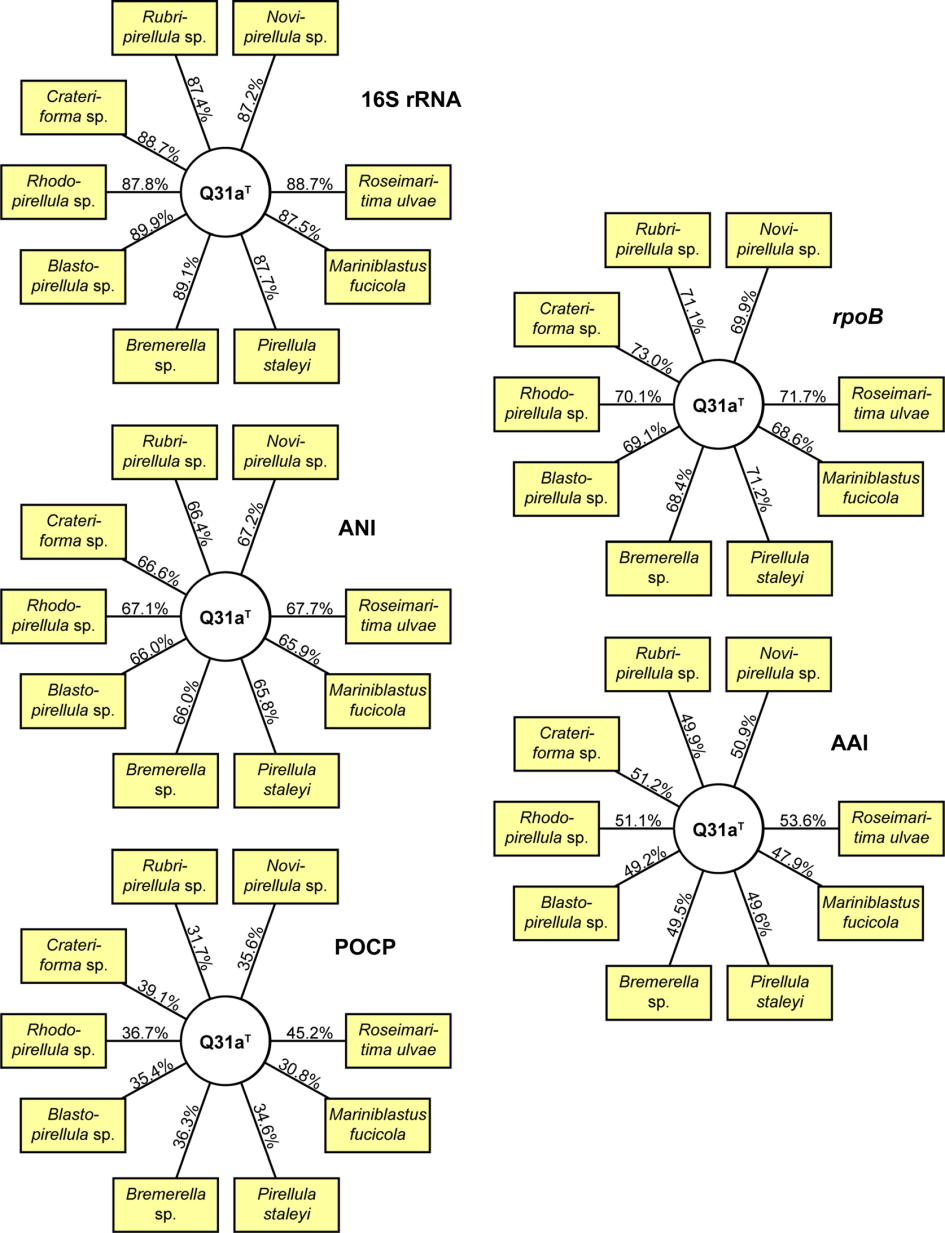
Phylogenetic marker values of Q31a^T^ and its current close neighbours. The numbers give the minimal similarity values shared between Q31a^T^ and any described member of the respective genera (angular boxes) for (16S rRNA) 16S rRNA gene identity, (*rpoB*) *rpoB* nucleotide sequences identity, (ANI) average nucleotide identity, (AAI) average amino acid identity and (POCP) percentage of conserved proteins

Based on 16S rRNA gene phylogeny and whole genome-based MLSA, strain Q31a^T^ groups within the planctomycetal family *Pirellulaceae* (Fig. 1). Within this family, its current closest neighbours on 16S rRNA gene level are *Mariniblastus fucicola* and *Pirellula staleyi*, further close neighbours are *Blastopirellula sp*. and *Bremerella sp*. However, supporting bootstrap values in this clade are sometimes rather low and no definitive closest neighbourhood could be determined by MLSA analysis. Therefore, strain Q31a^T^ was compared to all described genera within the family *Pirellulaceae* (Fig. 2).

16S rRNA gene sequence identity analysis (Fig. 2) shows that all minimal identities between the novel strain Q31a^T^ and the nine most related genera are notably below the genus threshold of 94.5% that might place Q31a^T^ in any of these taxa (Yarza et al. 2014). The similarity values for strain Q31a^T^ and its relatives are also below the genus threshold values used with *rpoB* nucleotide sequences identities (75.5–78.0%) (Kallscheuer et al. 2019d), AAI (60–80%) (Konstantinidis and Tiedje 2005) and POCP (50%) (Qin et al. 2014) (Fig. 2). With all used methods suggesting the placement of strain Q31a^T^ in a novel genus, we conclude the strain represents a novel genus and species within the family *Pirellulaceae*, for which we propose the name *Aureliella helgolandensis* gen. nov., sp. nov.

### Morphological and physiological analyses

**Fig. 3:**
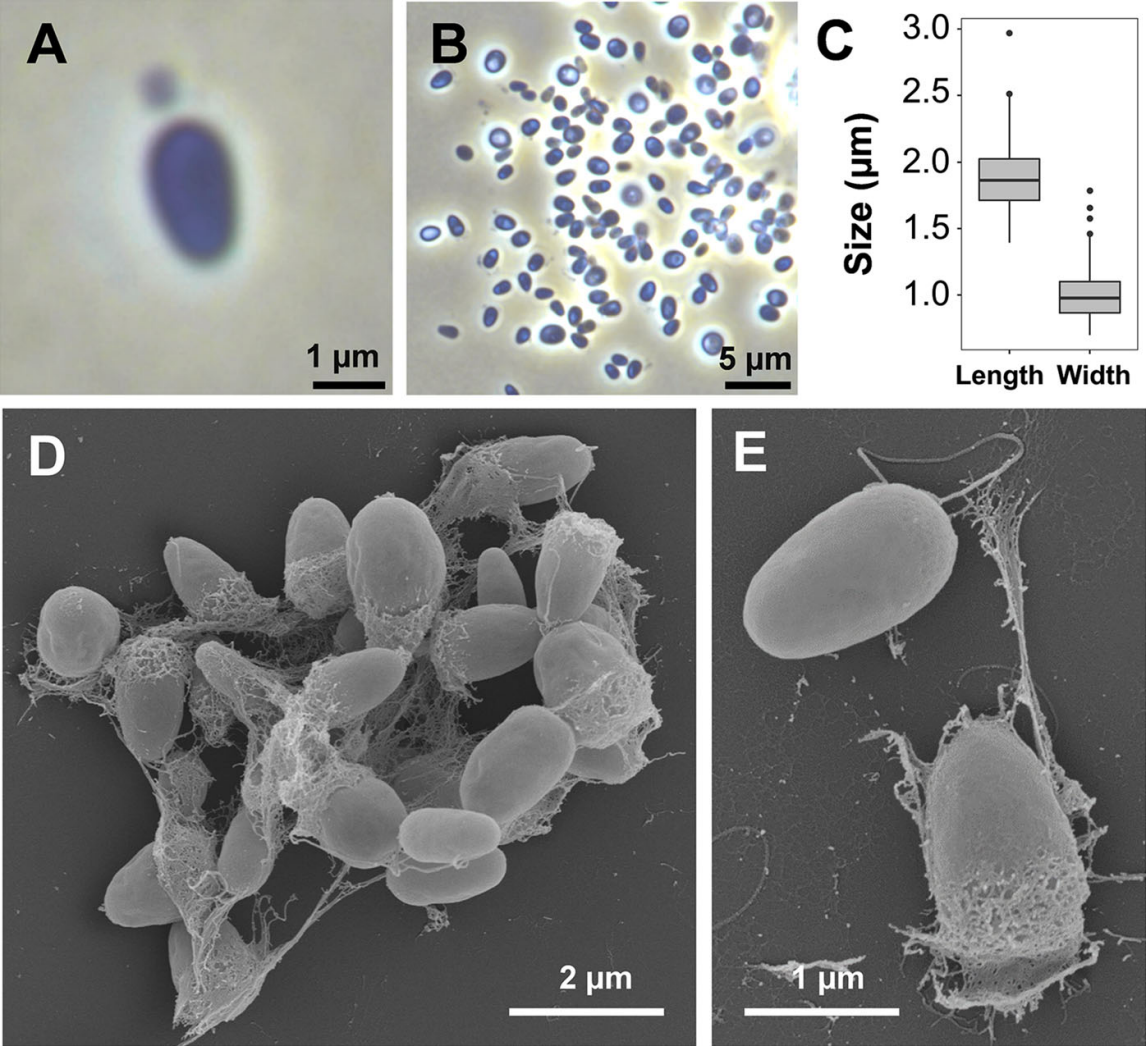
Microscopy images and cell size plot of strain Q31a^T^. The mode of cell division (**a**) and a general overview of cell morphology (**b**, **d**, **e**) are shown in the pictures. For determination of the cell size (**c**) at least 100 representative cells were counted manually or by using a semi-automated object count tool

Basic features of strain Q31a^T^ regarding cell morphology and mechanism of cell division are summarised in Table 1. As we could not identify a current closest relative of Q31a^T^, the morphological and genomic features were compared to all strains identified as potential candidates during the phylogenetic analysis. Morphological features of Q31a^T^ cells harvested during the exponential growth phase were analysed using phase contrast and scanning electron microscopy (Fig. 3). Cells of strain Q31a^T^ are 1.9 ± 0.2 × 1.0 ± 0.2 µm in size and acorn-shaped (Fig. 3a–c). Cells form aggregates of typically 8–25 cells, which in turn are often loosely connected to each other (Fig. 3d). Cells divide by polar budding (Fig. 3a). Extracellular matrix or fibre originates from one pole. At this pole crateriform structures can also be observed, which cover around 10–20% of the cell surface. Daughter cells of Q31a^T^ have the same shape as the mother cell. The strain follows a dimorphic lifecycle involving sessile mother cells and flagellated daughter cells. Colonies of strain Q31a^T^ lack pigmentation and have a lucid white colour.

**Table 1:** Phenotypic and genotypic features of strain Q31a^T^ compared to closely related strains

	Q31a^T^	*M. fucicola* FC18^T^	*B. marina* DSM 3645^T^	*R. ulvae* UC8^T^	*P. staleyi* DSM 6068^T^	*C. conspicua* Mal65^T^
Phenotypic characteristics						
Shape	Acorn-shaped	Spherical to ovoid	Ovoid, pear-shaped	Spherical to ovoid	Ovoid to spherical	Pear-shaped
Size length (µm)	1.9 ± 0.2	1.0–2.0 (diameter)	1.0–2.0	1.1–1.8	1.0–1.5	1.8 ± 0.3
Size width (µm)	1.0 ± 0.2	1.0–2.0 (diameter)	0.7–1.5	0.9–1.5	0.9-1.0	1.0 ± 0.2
Colony colour	Lucid white	White to light pink	Off-white to light brown	Light pink	Yellowish-white	Pink
Temperature range (optimum) (°C)	10–33 (27)	10–30 (25)	Up to 35 (27–33)	15–35 (30)	18–30 (24)	10–36 (32)
pH range (optimum)	6.0–8.0 (7.5)	6.5–8.5 (7.5)	5.5–8.5 (6.5–7.5)	6.5–10.0 (7.5)	n.d.	5.0–10.0 (7.5)
Aggregates	Yes	Yes	Yes	Yes, rosettes	Yes, rosettes	Yes, rosettes
Division	Budding	Budding	Budding	Budding	Budding	Budding
Dimorphic life cycle	Yes	No	Yes	Yes	Yes	Yes
Flagella	Yes	No	Yes	Yes	Yes	Yes
Crateriform structures	Yes, at fibre pole	Yes, at reproductive pole	Yes, at reproductive pole	Yes, at reproductive pole	Yes, at reproductive pole	Yes, at fibre pole
Fimbriae	Yes	Yes	Yes	Yes	Yes	Yes
Bud shape	Like mother cell	Like mother cell	Bean-shaped	Like mother cell	Like mother cell	Like mother cell
Budding type	Polar	Polar	Polar	Polar	Polar	Polar
Holdfast structure	Yes	Yes	Yes	Yes	Yes	No
Genomic characteristics						
Genome size (bp)	8,439,957	6,570,840	6,663,851	8,212,515	6,196,199	7,182,433
Plasmids (bp)	No	No	n.d.	No	No	No
G + C (%)	55.3	53.4	57.0	59.1	57.5	57.8
Completeness (%)	98.12	98.28	96.55	98.28	98.28	95.69
Contamination (%)	1.72	1.72	1.72	1.72	1.72	0
Protein-coding genes	6419	5123	5406	5815	4705	5437
Hypothetical proteins	2988	2087	3023	2299	2601	2117
Protein-coding genes/Mb	761	780	777	708	720	757
Coding density (%)	85.0	88.8	86.8	87.5	86.2	88.5
tRNAs	82	65	56	71	49	45
16S rRNA	2	1	1	1	1	1

The genome analysis is based on GenBank accession numbers CP036298 (Q31a^T^), CP042912 (*Mariniblastus fucicola* FC18^T^), GCF_000153105 (*Blastopirellula marina* DSM 3645^T^), CP042914 (*Roseimaritima ulvae* UC8^T^), GCF_000025185 (*Pirellula staleyi* DSM 6068^T^) and CP036319 (*Crateriforma conspicua* Mal65^T^)

**Fig. 4:**
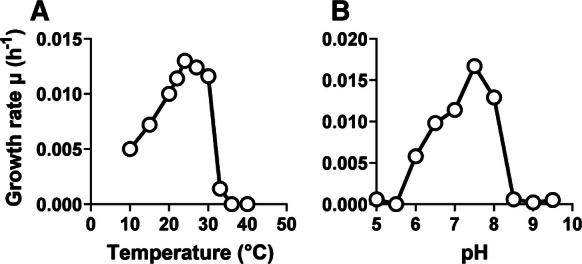
Temperature and pH optimum of Q31a^T^. The graphs show the average growth rates obtained from cultivation of Q31a^T^ in M1H NAG ASW medium in biological triplicates. Cultivations at different pH values were conducted at 28 °C and cultivations at different temperatures were performed at pH 7.5

Despite aggregate formation, measurement of optical densities (OD_600_) in liquid cultures was possible. In M1H NAG ASW medium, Q31a^T^ was found to be able to grow in a temperature range of 10–33 °C and in a pH range of 6.0–8.0 (Fig. 4). Optimal growth was observed at 27 °C and pH 7.5. The maximal growth rate observed in M1H NAG ASW medium was 0.017 h^− 1^ (Fig. 4), corresponding to a generation time of 41 h. Q31a^T^ is an aerobic heterotroph. During comparison of preferred temperature and pH of Q31a^T^ with the close relatives *M. fucicola* FC18^T^, *Blastopirellula marina* DSM 3645^T^, *Roseimaritima ulvae* UC8^T^, *P. staleyi* DSM 6068^T^ and *Crateriforma conspicua* Mal65^T^ considerable differences were observed. The temperature optimum of Q31a^T^ is between the optima of *P. staleyi* (24 °C)/*M. fucicola* (25 °C) and the other three strains (30–36 °C) (Table 1). The pH range for growth of Q31a^T^ is narrow compared to ranges of 6.0–10.0 observed for *R. ulvae* and *C. conspicua*, but comparable to *M. fucicola* (for the other two strains no data was available). The lucid white colony colour of Q31a^T^ indicates the lack of carotenoid formation of the strain, which is a common feature of *B. marina* and *P. staleyi*, but separates Q31a^T^ from the pink-pigmented *R. ulvae* and *C. conspicua* (Table 1).

### Genomic characteristics

The genome of Q31a^T^ has a size of 8.44 Mb and a G + C content of 55.3%. The strain lacks plasmids. The closed genome harbours 6419 protein-coding genes, almost half of which are annotated as hypothetical proteins (2988 genes, 47%). The number of protein-coding genes yields 761 genes per Mb and a coding density of 85.0%. 82 tRNAs are encoded and two copies of the 16S rRNA gene are present. In comparison to close relatives, Q31a^T^ has the largest genome and the highest number of protein-coding genes, tRNA- and 16S rRNA genes, but a lower G + C content (55% for Q31a^T^ vs. 57–59% for the other species, except *M. fucicola*, 53%). The number of protein-coding genes per Mb (710–780) is comparable for all six strains, however the coding density of 85% of Q31a^T^ is slightly lower compared to the other five species (87–89%).

### Genome-based analysis of metabolic features

Numbers of carbohydrate-active enzymes and secondary metabolite-associated genes clusters were analysed based on the genome sequences of Q31a^T^ and type species of related genera (Table 2). These numbers give a first impression on the potential of Q31a^T^ for degradation of complex and highly decorated polysaccharides and for production of bioactive small molecules. In total, Q31a^T^ harbours 159 carbohydrate-active enzyme as currently listed in the CAZY database. This number is comparable to *R. ulvae* UC8^T^, which also has a similar genome size. Although having a genome 1.3 Mb smaller than Q31a^T^, the highest number of 217 carbohydrate-active enzymes was observed in *C. conspicua* Mal65^T^. This difference can mainly be attributed to the glycoside hydrolase family, since 52 enzymes were found in Q31a^T^ and 121 in *C. conspicua* Mal65^T^. Q31a^T^ contains the highest number of enzymes of the carbohydrate esterase family of the species used for comparison.

**Table 2:** Numbers of carbohydrate-active enzymes and putative gene clusters involved in the production of secondary metabolites

	Q31a^T^	*M. fucicola* FC18^T^	*B. marina* DSM 3645^T^	*R. ulvae* UC8^T^	*P. staleyi* DSM 6068^T^	*C. conspicua* Mal65^T^
Genome size (Mb)	8.44	6.57	6.66	8.21	6.20	7.18
Carbohydrate-active enzymes						
Glycoside hydrolase family	52	44	n.d.	45	19	121
Glycosyltransferase family	73	57	n.d.	76	49	65
Polysaccharide lyase family	6	5	n.d.	3	1	7
Carbohydrate esterase family	12	5	n.d.	7	8	9
Carbohydrate-binding module family	16	15	n.d.	21	14	15
Total	159	126	n.d.	152	91	217
Putative secondary metabolite-associated genes clusters						
Terpene	2	2	3	2	3	2
Type I PKS	0	0	1	3	0	2
Type II PKS	0	0	0	0	0	0
Type III PKS	1	0	0	1	0	0
NRPS	0	0	0	0	0	1
Type I-PKS-NRPS	0	2	1	1	0	2
Bacteriocin	1	0	1	0	1	0
Ectoine	0	0	1	0	0	0
Resorcinol	1	0	0	0	0	0
Other	2	1	3	1	1	2
Total	7	5	10	8	5	9

The analysis is based on GenBank accession numbers CP036298 (Q31a^T^), CP042912 (*Mariniblastus fucicola* FC18^T^), GCF_000153105 (*Blastopirellula marina* DSM 3645^T^), CP042914 (*Roseimaritima ulvae* UC8^T^), GCF_000025185 (*Pirellula staleyi* DSM 6068^T^) and CP036319 (*Crateriforma conspicua* Mal65^T^). The genome of *B. marina* was not listed in the CAZY database

During analysis of secondary metabolite-associated gene clusters a heterogeneous distribution for the investigated species was observed. While 2–3 terpenoid-related clusters were found in all species, other clusters putatively involved in the production of ectoine, resorcinol or non-ribosomal peptides seem to be restricted to individual genera. Similar results were obtained for comparison of type I and type III polyketide synthases (PKSs), while type II PKSs appear to be absent from the compared genomes. The total numbers of predicted clusters is between 5 and 10, while higher numbers are not reflected by larger genomes in this case. Q31a^T^ has the largest genome, but is ranked 3rd with regard to the number of gene clusters. In contrast, *B. marina* DSM 3645^T^ has the highest number of predicted gene clusters, but is amongst the species with the smallest genomes.

## Conclusions

The performed comparison of morphological, physiological and genomic features supports the results of the phylogenetic analysis that Q31a^T^ does not belong to the genera *Mariniblastus*, *Pirellula*, *Blastopirellula*, *Rhodopirellula*, *Novipirellula*, *Rubripirellula*, *Bremerella*, *Crateriforma* or *Roseimaritima*, but instead represents a new species belonging to a novel genus. Thus, we propose the name *Aureliella helgolandensis* gen. nov., sp. nov. for Q31a^T^ and propose this species as the type species of the genus and Q31a^T^ as the type strain of the novel species.

### ***Aureliella*gen. nov**.

*Aureliella* (Au.re.li.el’la. N.L. fem. n. *Aureliella* dim. of Aurelia; a bacterium isolated from the common jellyfish *Aurelia aurita*).

Members of the genus are Gram-negative, aerobic, mesophilic, neutrophilic and heterotrophic. Cells are acorn-shaped, have crateriform structures at one pole and divide by polar budding. The genus belongs to the family *Pirellulaceae*, order *Pirellulales*, class *Planctomycetia*, phylum *Planctomycetes*. The type species is *Aureliella helgolandensis*.

### ***Aureliella helgolandensis*sp. nov**.

*Aureliella helgolandensis* (hel.go.lan.den’sis N.L. fem. adj. *helgolandensis* of Helgoland; corresponding to the origin of the strain from the German island Helgoland).

Cells are 1.9 ± 0.2 µm × 1.0 ± 0.2 µm in size and form aggregates. Matrix or fibre originates from the budding pole and a holdfast structure is present at the opposite pole. Grows at 10–33 °C (optimum 27 °C) and at pH 6.0–8.0 (optimum 7.5). Colonies are lucid white. The genome of the type strain has a G + C content of 55.3%.

The type strain is Q31a^T^ (DSM 103537^T^ = LMG 29700^T^), isolated from a jellyfish (*Aurelia aurita*) on the shore of Helgoland Island in June 2013. The type strain genome (8.44 Mb, acc. no. CP036298) and 16S rRNA gene sequence (acc. no. MK559992) are available from GenBank.
